# On new common fixed point theorems via bipolar fuzzy b-metric space with their applications

**DOI:** 10.1371/journal.pone.0305316

**Published:** 2024-06-25

**Authors:** J. Uma Maheswari, K. Dillibabu, Gunaseelan Mani, Sabri T. M. Thabet, Imed Kedim, Miguel Vivas-Cortez

**Affiliations:** 1 PG and Research Department of Mathematics, St. Joseph’s College, Affiliated to Bharathidasan University, Trichy, Tamil Nadu, India; 2 Department of Mathematics, Sir Theagaraya College, Chennai, Tamil Nadu, India; 3 Department of Mathematics, Saveetha School of Engineering, Saveetha Institute of Medical and Technical Sciences, Saveetha University, Chennai, Tamil Nadu, India; 4 Department of Mathematics, Radfan University College, University of Lahej, Lahej, Yemen; 5 Department of Mathematics, College of Science, Korea University, Seoul, South Korea; 6 Department of Mathematics, College of Science and Humanities in Al-Kharj, Prince Sattam Bin Abdulaziz University, Al-Kharj, Saudi Arabia; 7 Faculty of Exact and Natural Sciences, School of Physical Sciences and Mathematics, Pontifical Catholic University of Ecuador, Sede Quito, Ecuador; Korea National University of Transportation, KOREA, REPUBLIC OF

## Abstract

This research work is devoted to investigating new common fixed point theorems on bipolar fuzzy b-metric space. Our main findings generalize some of the existence outcomes in the literature. Furthermore, we illustrate our findings by providing some applications for fractional differential and integral equations.

## 1 Introduction and basic materials

Sklar and Schweizer first developed a continuous triangular norm [1] in 1960. Following that, Zadeh [2] presented the fuzzy set theory in 1965. Michalek and Kramosil [3] introduced the fuzzy metric space (FMS) in 1975 using the idea of fuzziness and the continuous t-norm. The fuzzy concept to distance is based on the assumption that the distance between any two points, which we must estimate or find, need not always be defined by a real number; rather, it is a fuzzy concept. Veeramani and George [4] updated the FMSs definition in 1994. Karamosil and Michalek [3], Grabeic [5] extended the Banach fixed point theorem to FMSs. The fuzzy Banach contraction theorem was extended to FMS by Gregori and Sapena [6] in the sense of George and Veeramani’s [4]. Roy, Saha, George, Gurand and Mitrović [7] introduced bipolar p-metric spaces and proved fixed point theorems. Fixed point results have been established in the setting of bipolar metric space by Karapınar and M. Cvetković [8]. It examined into how these results related to analogous fixed point results in metric space, ultimately obtaining equivalency. Concepts of Pompeiu-Hausdorff bipolar metric, multivalued covariant, and contravariant contraction mappings in bipolar metric spaces were introduced by Mutlu, Özkan, and Gürdal [9]. Bipolar metric spaces, as generalized by Mutlu and Gurdal [10], provide a novel framework for measuring the farness between component of distinct two sets. Recently, fixed point theorems were proven, and fuzzy bipolar metric space was presented by Bartwal et al. [11]. Bipolar controlled fuzzy metric spaces were first proposed by Tiwari and Rajput [12], which also demonstrated fixed point theorems. Further articles which relate can be seen [13–18]. Ramalingam et al. [19], presented the fuzzy bipolar *b*-metric space in 2023 and used the triangular property to prove some fixed point theorems without continuity.

In this research article, we prove some new common fixed point (CFP) theorems on bipolar fuzzy b-metric space (BFBMS) with applications on fractional differential and integral equations.

Now, we present some basic definitions, lemmas and propositions as follows:

**Definition 1.1**. [4] *Let Γ be a non-void set. An three tuple* (*Γ*, *Ω*, *) *is called a FMS, if Ω is a fuzzy set on Γ*^2^ × (0, ∞), and * is a continuous π-norm such that ∀ *φ*, *ϖ*, *ϕ* ∈ *Γ*, *and* *π*, ℧ > 0;

1. *Ω*(*φ*, *ϖ*, *π*) > 0;
2. *Ω*(*φ*, *ϖ*, *π*) = 1 *iff* *φ* = *ϖ*;
3. *Ω*(*φ*, *ϖ*, *π*) = *Ω*(*ϖ*, *φ*, *π*);
4.
Ω(φ,ϕ,t+s)≥Ω(φ,ϖ,t)*Ω(ϖ,ϕ,s) ;
5. *Ω*(*φ*, *ϖ*, .) : (0, ∞) → (0, 1] *is continuous*.

**Definition 1.2**. [19] *Let* Φ *and Γ be two non-void sets and* ℧ ≥ 1 *be a given real number. A five tuple* $\left(\Phi ,\Gamma ,\Omega_{b},℧,*\right)$ *is called BFBMS, where* $\Omega_{b}$ *is a fuzzy set on* Φ × *Γ* × (0, ∞), and * is continuous π-norm, satisfying ∀π,℧,r>0:

*(FB1)* $\Omega_{b}\left(\varphi ,\varpi ,\pi \right)>0\;\forall \;\left(\varphi ,\varpi \right)\in \Phi \times \Gamma$ ;
*(FB2)* $\Omega_{b}\left(\varphi ,\varpi ,\pi \right)=1$ *iff* *φ* = *ϖ for φ* ∈ Φ *and ϖ* ∈ *Γ*;
*(FB3)* $\Omega_{b}\left(\varphi ,\varpi ,\pi \right)=\Omega_{b}\left(\varpi ,\varphi ,\pi \right)\;\forall \;\varphi ,\varpi \in \Phi \cap \Gamma$ ;
*(FB4)* $\Omega_{b}\left(\varphi_{1},\varpi_{2},℧\left(t+s+r\right)\right)\geq \Omega_{b}\left(\varphi_{1},\varpi_{1},t\right)*\Omega_{b}\left(\varphi_{2},\varpi_{1},s\right)*\Omega_{b}\left(\varphi_{2},\varpi_{2},r\right)$ ∀ *φ*_1_, *φ*_2_ ∈ Φ *and ϖ*_1_, *ϖ*_2_ ∈ *Γ*;
*(FB5)* $\Omega_{b}\left(\varphi ,\varpi ,.\right):\left[0,\infty \right)\to \left[0,1\right]$ *is left continuous*;
*(FB6)* $\Omega_{b}\left(\varphi ,\varpi ,.\right)$ *is non-decreasing* ∀ *φ* ∈ Φ *and ϖ* ∈ *Γ*.

**Example 1.1**. *Let* (Φ,Γ,d) *be an BFBMS. For all φ* ∈ Φ, *ϖ* ∈ *Γ*, *and π* > 0, *denote*

$$\begin{array}{c} \Omega_{b}\left(\varphi ,\varpi ,\pi \right)=\frac{\pi }{\pi +{\left(d\left(\varphi ,\varpi \right)\right)}^{2}}. \end{array}$$

*Then* $\left(\Phi ,\Gamma ,\Omega_{b},℧,*\right)$ *is a BFBMS, where is a π-norm given by* i*b=ib *or* i*b=min{i,b}.

**Definition 1.3**. [19] *Assume that a BFBMS is* $\left(\Phi ,\Gamma ,\Omega_{b},℧,*\right)$. Φ, *Γ and* Φ ∩ *Γ have points that are referred to as left, right, and central points, respectively, and* Φ, *Γ and* Φ ∩ *Γ have sequences that are referred to as left, right, and central sequences, respectively*.

**Lemma 1.1**. *Let* $\left(\Phi ,\Gamma ,\Omega_{b},℧,*\right)$ *be a BFBMS such that (s.t.)*

$$\begin{array}{c} \Omega_{b}\left(\varphi ,\varpi ,\sigma \pi \right)\geq \Omega_{b}\left(\varphi ,\varpi ,\pi \right) \end{array}$$

*for φ* ∈ Φ, *ϖ* ∈ *Γ and σ* ∈ (0, 1). *Then φ* = *ϖ*.

*Proof*. One has

$$\begin{array}{c} \Omega_{b}\left(\varphi ,\varpi ,\sigma \pi \right)\geq \Omega_{b}\left(\varphi ,\varpi ,\pi \right)\;\text{for}\;\pi >0. \end{array}$$ (1)

Since *σπ* < *π* ∀ *π* > 0 and *σ* ∈ (0, 1), by (FB6) we have

$$\begin{array}{c} \Omega_{b}\left(\varphi ,\varpi ,\sigma \pi \right)\leq \Omega_{b}\left(\varphi ,\varpi ,\pi \right). \end{array}$$ (2)

From (1) and (2), we get *φ* = *ϖ*.

**Definition 1.4**. [19] *Let* $\left(\Phi ,\Gamma ,\Omega_{b},℧,*\right)$ *be a BFBMS. A sequence* {*φ*_*ν*_} ∈ Φ *converges to a right point ϖ iff for each ϵ* > 0 *and π* > 0, *we can find* $\nu_{0}\in N$ *s.t*. $\Omega_{b}\left(\varphi_{\nu },\varpi ,\pi \right)>1-\epsilon \;\forall \;\nu \geq \nu_{0}$, *i.e*., $\Omega_{b}\left(\varphi_{\nu },\varpi ,\pi \right)\to 1$ *as ν* → ∞ ∀ *π* > 0, *that can be represented mathematically as* {*φ*_*ν*_} → *ϖ or* lim_*ν*→∞_ *φ*_*ν*_ = *ϖ* *as ν* → ∞. *Likewise, a right sequence* {*ϖ*_*ν*_} *converges to a left point φ iff for every ϵ* > 0 *and π* > 0, *we can find* $\nu_{0}\in N$ *s.t*. $\Omega_{b}\left(\varphi ,\varpi_{\nu },\pi \right)>1-\epsilon \;\forall \;\nu \geq \nu_{0}$, *i.e*., $\Omega_{b}\left(\varphi ,\varpi_{\nu },\pi \right)\to 1$ *as ν* → ∞ ∀ *π* > 0, *that can be represented mathematically as* {*ϖ*_*ν*_} → *φ* *or* lim_*ν*→∞_ *ϖ*_*ν*_ = *φ* *as ν* → ∞).

**Definition 1.5**. [19] *Let* $\left(\Phi ,\Gamma ,\Omega_{b},℧,*\right)$ *be a BFBMS then*:

*(i) Sequence or Bisequence* ({*φ*_*ν*_}, {*ϖ*_*ν*_}) ∈ Φ × *Γ is named as a bisequence on* $\left(\Phi ,\Gamma ,\Omega_{b},℧,*\right)$.
*(ii) The sequence or bisequence* ({*φ*_*ν*_}, {*ϖ*_*ν*_}) ∈ Φ × *Γ* *is said to be convergent if both* {*φ*_*ν*_} and {*ϖ*_*ν*_} *converge. A bisequence* (*φ*_*ν*_, *ϖ*_*ν*_) *is referred to as a biconvergent sequence if both* {*φ*_*ν*_} *and* {*ϖ*_*ν*_} *converge to a center point*.
*(iii) A bisequence* ({*φ*_*ν*_}, {*ϖ*_*ν*_}) *on BFBMS* $\left(\Phi ,\Gamma ,\Omega_{b},℧,*\right)$ *is said to be a Cauchy bisequence if for each ϵ* > 0, *we can find* $\nu_{0}\in N$ *s.t. for all* $\nu ,\kappa \geq \nu_{0}\left(\nu ,\kappa \in N\right)$, *we have* $\Omega_{b}\left(\varphi_{\nu },\varpi_{\kappa },\pi \right)>1-\epsilon$ *for each π* > 0, *i.e., a bisequence* ({*φ*_*ν*_}, {*ϖ*_*ν*_}) *is said to be a Cauchy bisequence if* $\Omega_{b}\left(\varphi_{\nu },\varpi_{\kappa },\pi \right)\to 1$ *as ν*, *κ* → ∞ *for all π* > 0.

**Definition 1.6**. [19] *If each Cauchy bisequence in* Φ × *Γ* *is biconvergent in* Φ × *Γ*, *the BFBMS* $\left(\Phi ,\Gamma ,\Omega_{b},℧,*\right)$ *is regarded as complete*.

**Proposition 1.1**. [19] *In a BFBMS, every convergent Cauchy bisequence is biconvergent*.

**Definition 1.7**. *A space φ* ∈ Φ ∩ *Γ is said to be CFP for the mappings* (T,S) *on φ* ∈ Φ ∩ *Γ s.t*. φ=Tφ=Sφ.

## 2 Main results

This section begins with the common fixed point theorem on BFBMS under covariant mappings.

**Theorem 2.1**. *Let* $\left(\Phi ,\Gamma ,\Omega_{b},℧,*\right)$ *be a complete BFBMS s.t*.

$$\begin{array}{c} \underset{\pi \to \infty }{\text{lim}}\;\Omega_{b}\left(\varphi ,\varpi ,\pi \right)=1\;\forall \;\varphi \in \Phi ,\varpi \in \Gamma . \end{array}$$ (3)

*Let* T,S:Φ∪Γ→Φ∪Γ *be mapping satisfying*

*(i)* T(Φ)⊆Φ , S(Φ)⊆Φ *and* T(Γ)⊆Γ, S(Γ)⊆Γ;
*(ii)* $\Omega_{b}\left(T\left(\varphi \right),S\left(\varpi \right),\sigma \pi \right)\geq \Omega_{b}\left(\varphi ,\varpi ,\pi \right)\;\forall \;\varphi \in \Phi ,\varpi \in \Gamma$ , *and π* > 0, *whenever σ* ∈ (0, 1).

*Then*, T *and* S *admits a unique CFP (UCFP)*.

*Proof*. Fix *φ*_0_ ∈ Φ and *ϖ*_0_ ∈ *Γ* and assume that $T\left(\varphi_{2\nu }\right)=\varphi_{2\nu +1}$, $S\left(\varphi_{2\nu +1}\right)=\varphi_{2\nu +2}$ and $T\left(\varpi_{2\nu }\right)=\varpi_{2\nu +1}$, $S\left(\varpi_{2\nu +1}\right)=\varpi_{2\nu +2}\;\forall \;\nu \in N\cup \left\{0\right\}$. Then ({*φ*_*ν*_}, {*ϖ*_*ν*_}) is a bisequence on BFBMS $\left(\Phi ,\Gamma ,\Omega_{b},℧,*\right)$. Now,

$$\begin{array}{c} \Omega_{b}\left(\varphi_{1},\varpi_{1},\pi \right)=\Omega_{b}\left(T\left(\varphi_{0}\right),S\left(\varpi_{0}\right),\pi \right)\geq \Omega_{b}\left(\varphi_{0},\varpi_{0},\frac{\pi }{\sigma }\right) \end{array}$$

for all *π* > 0 and ν∈N. Then

$$\begin{array}{c} \Omega_{b}\left(\varphi_{2\nu +1},\varpi_{2\nu +1},\pi \right)=\Omega_{b}\left(T\left(\varphi_{2\nu }\right),S\left(\varpi_{2\nu }\right),\pi \right)\geq \Omega_{b}\left(\varphi_{0},\varpi_{0},\frac{\pi }{\sigma^{2\nu +1}}\right) \end{array}$$ (4)

and

$$\begin{array}{c} \Omega_{b}\left(\varphi_{2\nu +1},\varpi_{2\nu },\pi \right)=\Omega_{b}\left(T\left(\varphi_{2\nu }\right),S\left(\varpi_{2\nu -1}\right),\pi \right)\geq \Omega_{b}\left(\varphi_{1},\varpi_{0},\frac{\pi }{\sigma^{2\nu }}\right) \end{array}$$ (5)

for all *π* > 0 and ν∈N.

Letting *ν* < *κ*, for ν,κ∈N. Then

$$\begin{array}{cc} \Omega_{b}\left(\varphi_{\nu },\varpi_{\kappa },\pi \right)\geq & \Omega_{b}\left(\varphi_{\nu },\varpi_{\nu },\frac{\pi }{3℧}\right)⋆\Omega_{b}\left(\varphi_{\nu +1},\varpi_{\nu },\frac{\pi }{3℧}\right)⋆\Omega_{b}\left(\varphi_{\nu +1},\varpi_{\kappa },\frac{\pi }{3℧}\right)\\ & \vdots \\ & \geq \Omega_{b}\left(\varphi_{\nu },\varpi_{\nu },\frac{\pi }{3℧}\right)⋆\Omega_{b}\left(\varphi_{\nu +1},\varpi_{\nu },\frac{\pi }{3℧}\right)⋆\cdots ⋆\Omega_{b}\left(\varphi_{\kappa -1},\varpi_{\kappa -1},\frac{\pi }{3^{\kappa -1}{℧}^{\kappa -1}}\right)\\ & ⋆\Omega_{b}\left(\varphi_{\kappa },\varpi_{\kappa -1},\frac{\pi }{3^{\kappa -1}{℧}^{\kappa -1}}\right)⋆\Omega_{b}\left(\varphi_{\kappa },\varpi_{\kappa },\frac{\pi }{3^{\kappa -1}{℧}^{\kappa -1}}\right). \end{array}$$

Consequently,

$$\begin{array}{cc} \Omega_{b}\left(\varphi_{\nu },\varpi_{\kappa },\pi \right) & \geq \Omega_{b}\left(\varphi_{0},\varpi_{0},\frac{\pi }{3\sigma^{\nu }℧}\right)⋆\Omega_{b}\left(\varphi_{1},\varpi_{0},\frac{\pi }{3\sigma^{\nu }℧}\right)⋆\cdots \\ & \cdots ⋆\Omega_{b}\left(\varphi_{0},\varpi_{0},\frac{\pi }{3^{\kappa -1}\sigma^{\kappa }{℧}^{\kappa -1}}\right). \end{array}$$

Letting *ν*, *κ* → ∞, we get

$$\begin{array}{c} \Omega_{b}\left(\varphi_{\nu },\varpi_{\kappa },\pi \right)\geq 1\;\forall \;\pi >0. \end{array}$$

Therefore, the bisequence ({*φ*_*ν*_}, {*ϖ*_*ν*_}) is a Cauchy bisequence. Since $\left(\Phi ,\Gamma ,\Omega_{b},℧,*\right)$ is a complete, the bisequence ({*φ*_*ν*_}, {*ϖ*_*ν*_}) is a biconvergent. Therefore, bisequence ({*φ*_*ν*_}, {*ϖ*_*ν*_}) is biconvergent then

$$\begin{array}{c} \left\{\varphi_{\nu }\right\}\to \upsilon \;\;\text{and}\;\;\left\{\varpi_{\nu }\right\}\to \upsilon \;\forall \;\upsilon \in \Phi \cap \Gamma . \end{array}$$

From (FB4),

$$\begin{array}{cc} \Omega_{b}\left(T\left(\upsilon \right),\upsilon ,\pi \right) & \geq \Omega_{b}\left(T\left(\upsilon \right),\varpi_{\nu +1},\frac{\pi }{3℧}\right)⋆\Omega_{b}\left(\varphi_{\nu +1},\varpi_{\nu +1},\frac{\pi }{3℧}\right)*\Omega_{b}\left(\varphi_{\nu +1},\upsilon ,\frac{\pi }{3℧}\right)\\ & =\Omega_{b}\left(T\left(\upsilon \right),S\left(\varpi_{\nu }\right),\frac{\pi }{3℧}\right)⋆\Omega_{b}\left(\varphi_{\nu +1},\varpi_{\nu +1},\frac{\pi }{3℧}\right)*\Omega_{b}\left(\varphi_{\nu +1},\upsilon ,\frac{\pi }{3℧}\right)\\ & \geq \Omega_{b}\left(\upsilon ,\varpi_{\nu },\frac{\pi }{3℧}\right)⋆\Omega_{b}\left(\varphi_{\nu +1},\varpi_{\nu +1},\frac{\pi }{3℧}\right)*\Omega_{b}\left(\varphi_{\nu +1},\upsilon ,\frac{\pi }{3℧}\right), \end{array}$$

∀ν∈N, *π* > 0, and as *ν* → ∞, one has

$$\begin{array}{c} \Omega_{b}\left(T\left(\upsilon \right),\upsilon ,\pi \right)\to 1*1*1=1. \end{array}$$

By (FB2), one gets T(υ)=υ. Again,

$$\begin{array}{cc} \Omega_{b}\left(\upsilon ,S\left(\upsilon \right),\pi \right) & =\Omega_{b}\left(T\left(\upsilon \right),S\left(\upsilon \right),\pi \right)\\ & \geq \Omega_{b}\left(\upsilon ,\upsilon ,\frac{\pi }{\sigma }\right)=1. \end{array}$$

Therefore, S(υ)=υ. Hence *υ* is CFP of T and S. Let *ρ* ∈ Φ ∩ *Γ* is another fixed point of T and S. Then

$$\begin{array}{c} \Omega_{b}\left(\upsilon ,\rho ,\pi \right)=\Omega_{b}\left(T\left(\upsilon \right),S\left(\rho \right),\pi \right)\geq \Omega_{b}\left(\upsilon ,\rho ,\frac{\pi }{\sigma }\right) \end{array}$$

for *σ* ∈ (0, 1) and ∀ *π* > 0. Therefore *υ* = *ρ*.

**Example 2.1**. *Assume that* Φ = [0, 2], *and* Γ={0}∪N-{1,2}. *Let us define* $\Omega_{b}=\frac{\pi }{{\pi +|\varphi -\varpi |}^{2}}\;\forall \;\pi >0$ *and φ* ∈ Φ, *ϖ* ∈ *Γ*. *Clearly*, $\left(\Phi ,\Gamma ,\Omega_{b},℧,*\right)$ is a complete BFBMS, where * is a continuous *π-norm given by* i*b=ib. *Define* T,S:Φ∪Γ→Φ∪Γ *given by*

$$\begin{array}{c} T\left(\varphi \right)=\{\begin{array}{cc} 2-\upsilon , & if\;\varphi \in [0,2],\\ 2, & if\;\varphi \in N-\{1,2\}, \end{array} \end{array}$$

*and*

$$\begin{array}{c} S\left(\varphi \right)=\{\begin{array}{cc} \varphi , & if\;\varphi \in [0,2],\\ 2, & if\;\varphi \in N-\{1,2\}, \end{array} \end{array}$$

*for all φ* ∈ Φ ∪ *Γ. Clearly*, T *and* S *are satisfied the axiom (i) of Theorem 2.1. Let* $\sigma =\frac{1}{2}$, *then* ∀ *π* > 0, *we obtain*

*Case 1: Let φ* ∈ [0, 2] *and ϖ* ∈ [0, 2], *then*
  $$\begin{array}{cc} \Omega_{b}\left(T\left(\varphi \right),S\left(\varpi \right),\sigma \pi \right) & =\Omega_{b}\left(2-\varphi ,\varpi ,\sigma \pi \right)\\ & =\frac{\sigma \pi }{{\sigma \pi +|2-\varphi -\varpi |}^{2}}\\ & \geq \frac{\pi }{{\pi +|\varphi -\varpi |}^{2}}\\ & =\Omega_{b}\left(\varphi ,\varpi ,\pi \right). \end{array}$$
*Case 2: If φ* ∈ [0, 2] *and* ϖ∈N-{1,2}, *then*
  $$\begin{array}{cc} \Omega_{b}\left(T\left(\varphi \right),S\left(\varpi \right),\sigma \pi \right) & =\Omega_{b}\left(2-\varphi ,2,\sigma \pi \right)\\ & =\frac{\sigma \pi }{{\sigma \pi +|2-\varphi -2|}^{2}}\\ & \geq \frac{\pi }{{\pi +|\varphi -\varpi |}^{2}}\\ & =\Omega_{b}\left(\varphi ,\varpi ,\pi \right). \end{array}$$
*Case 3: For* φ∈N-{1,2} *and ϖ* ∈ [0, 2], *then*
  $$\begin{array}{cc} \Omega_{b}\left(T\left(\varphi \right),S\left(\varpi \right),\sigma \pi \right) & =\Omega_{b}\left(2,\varpi ,\sigma \pi \right)\\ & =\frac{\sigma \pi }{{\sigma \pi +|2-\varpi |}^{2}}\\ & \geq \frac{\pi }{{\pi +|\varphi -\varpi |}^{2}}\\ & =\Omega_{b}\left(\varphi ,\varpi ,\pi \right). \end{array}$$
*Case 4: Whenever* φ∈N-{1,2} *and* ϖ∈N-{1,2} , *then*
  $$\begin{array}{cc} \Omega_{b}\left(T\left(\varphi \right),S\left(\varpi \right),\sigma \pi \right) & =\Omega_{b}\left(2,2,\sigma \pi \right)\\ & =1\\ & \geq \frac{\pi }{{\pi +|\varphi -\varpi |}^{2}}\\ & =\Omega_{b}\left(\varphi ,\varpi ,\pi \right). \end{array}$$
  *Therefore, axiom (ii) of Theorem 2.1 also fulfills by* T *and* S. *According to Theorem 2.1, one finds that* T *and* S *have a UCFP, i.e., φ* = 1.

Next, under contravariant mappings, the common fixed point theorem on BFBMS is presented.

**Theorem 2.2**. *Consider the complete BFBMS* $\left(\Phi ,\Gamma ,\Omega_{b},*\right)$ *to be s.t*.

$$\begin{array}{c} \underset{\pi \to \infty }{\text{lim}}\;\Omega_{b}\left(\varphi ,\varpi ,\pi \right)=1\;\forall \;\varphi \in \Phi ,\varpi \in \Gamma . \end{array}$$ (6)

*Give a mapping satisfying* T,S:Φ∪Γ→Φ∪Γ

*(i)* T(Φ)⊆Γ , T(Γ)⊆Φ *and* S(Φ)⊆Γ, S(Γ)⊆Φ;
*(ii)* $\Omega_{b}\left(T\left(\varpi \right),S\left(\varphi \right),\sigma \pi \right)\geq \Omega_{b}\left(\varphi ,\varpi ,\pi \right)\;\forall \;\varphi \in \Phi ,\varpi \in \Gamma$ *and* *π* > 0, *where σ* ∈ (0, 1).

*Then* T *and* S *have a UCFP*.

*Proof*. Fix *φ*_0_ ∈ Φ and *ϖ*_0_ ∈ *Γ* and assume that $T\left(\varphi_{2\nu }\right)=\varpi_{2\nu }$, $S\left(\varphi_{2\nu +1}\right)=\varpi_{2\nu +1}$ and $T\left(\varpi_{2\nu }\right)=\varphi_{2\nu +1}$, $S\left(\varpi_{2\nu +1}\right)=\varphi_{2\nu +2}\;\forall \;\nu \in N\cup \left\{0\right\}$. Then ({*φ*_*ν*_}, {*ϖ*_*ν*_}) is a bisequence on BFBMS $\left(\Phi ,\Gamma ,\Omega_{b},*\right)$. Now,

$$\begin{array}{c} \Omega_{b}\left(\varphi_{1},\varpi_{0},\pi \right)=\Omega_{b}\left(T\left(\varpi_{0}\right),S\left(\varphi_{0}\right),\pi \right)\geq \Omega_{b}\left(\varphi_{0},\varpi_{0},\frac{\pi }{\sigma }\right) \end{array}$$

for all *π* > 0 and ν∈N. Then

$$\begin{array}{c} \Omega_{b}\left(\varphi_{2\nu +1},\varpi_{2\nu +1},\pi \right)=\Omega_{b}\left(T\left(\varpi_{2\nu }\right),S\left(\varphi_{2\nu +1}\right),\pi \right)\geq \Omega_{b}\left(\varphi_{0},\varpi_{0},\frac{\pi }{\sigma^{4\nu +2}}\right) \end{array}$$ (7)

and

$$\begin{array}{c} \Omega_{b}\left(\varphi_{2\nu +1},\varpi_{2\nu },\pi \right)=\Omega_{b}\left(T\left(\varpi_{2\nu }\right),S\left(\varphi_{2\nu }\right),\pi \right)\geq \Omega_{b}\left(\varphi_{0},\varpi_{0},\frac{\pi }{\sigma^{4\nu +1}}\right) \end{array}$$ (8)

for all *π* > 0 and ν∈N. Letting *ν* < *κ*, for ν,κ∈N. Then

$$\begin{array}{cc} \Omega_{b}\left(\varphi_{\nu },\varpi_{\kappa },\pi \right)\geq & \Omega_{b}\left(\varphi_{\nu },\varpi_{\nu },\frac{\pi }{3℧}\right)⋆\Omega_{b}\left(\varphi_{\nu +1},\varpi_{\nu },\frac{\pi }{3℧}\right)⋆\Omega_{b}\left(\varphi_{\nu +1},\varpi_{\kappa },\frac{\pi }{3℧}\right)\\ & \vdots \\ & \geq \Omega_{b}\left(\varphi_{\nu },\varpi_{\nu },\frac{\pi }{3℧}\right)⋆\Omega_{b}\left(\varphi_{\nu +1},\varpi_{\nu },\frac{\pi }{3℧}\right)⋆\cdots \\ & ⋆\Omega_{b}\left(\varphi_{\kappa -1},\varpi_{\kappa -1},\frac{\pi }{3^{\kappa -1}{℧}^{\kappa -1}}\right)⋆\Omega_{b}\left(\varphi_{\kappa },\varpi_{\kappa -1},\frac{\pi }{3^{\kappa -1}{℧}^{\kappa -1}}\right)\\ & ⋆\Omega_{b}\left(\varphi_{\kappa },\varpi_{\kappa },\frac{\pi }{3^{\kappa -1}{℧}^{\kappa -1}}\right). \end{array}$$

Consequently,

$$\begin{array}{cc} \Omega_{b}\left(\varphi_{\nu },\varpi_{\kappa },\pi \right) & \geq \Omega_{b}\left(\varphi_{0},\varpi_{0},\frac{\pi }{3\sigma^{2\nu }℧}\right)⋆\Omega_{b}\left(\varphi_{0},\varpi_{0},\frac{\pi }{3\sigma^{2\nu +1}℧}\right)⋆\cdots \\ & \cdots ⋆\Omega_{b}\left(\varphi_{0},\varpi_{0},\frac{\pi }{3^{\kappa -1}\sigma^{2\kappa }{℧}^{\kappa -1}}\right). \end{array}$$

Letting *ν*, *κ* → ∞, we get

$$\begin{array}{c} \Omega_{b}\left(\varphi_{\nu },\varpi_{\kappa },\pi \right)\geq 1\;\forall \;\pi >0. \end{array}$$

Thus, the bisequence ({*φ*_*ν*_}, {*ϖ*_*ν*_}) is a Cauchy bisequence. Since $\left(\Phi ,\Gamma ,\Omega_{b},℧,*\right)$ is a complete. Therefore, the bisequence ({*φ*_*ν*_}, {*ϖ*_*ν*_}) is biconvergent then

$$\begin{array}{c} \left\{\varphi_{\nu }\right\}\to \upsilon \;\;\text{and}\;\;\left\{\varpi_{\nu }\right\}\to \upsilon \;\forall \;\upsilon \in \Phi \cap \Gamma . \end{array}$$

From (FB4),

$$\begin{array}{cc} \Omega_{b}\left(T\left(\upsilon \right),\upsilon ,\pi \right) & \geq \Omega_{b}\left(T\left(\upsilon \right),\varpi_{\nu +1},\frac{\pi }{3℧}\right)⋆\Omega_{b}\left(\varphi_{\nu +1},\varpi_{\nu +1},\frac{\pi }{3℧}\right)*\Omega_{b}\left(\varphi_{\nu +1},\upsilon ,\frac{\pi }{3℧}\right)\\ & =\Omega_{b}\left(T\left(\upsilon \right),S\left(\varphi_{\nu +1}\right),\frac{\pi }{3℧}\right)⋆\Omega_{b}\left(T\varpi_{\nu },S\varphi_{\nu +1},\frac{\pi }{3℧}\right)⋆\Omega_{b}\left(\varphi_{\nu +1},\upsilon ,\frac{\pi }{3℧}\right)\\ & \geq \Omega_{b}\left(\upsilon ,\varphi_{\nu +1},\frac{\pi }{3\sigma ℧}\right)⋆\Omega_{b}\left(\varpi_{\nu },\varphi_{\nu +1},\frac{\pi }{3\sigma ℧}\right)*\Omega_{b}\left(\varphi_{\nu +1},\upsilon ,\frac{\pi }{3℧}\right), \end{array}$$

∀ν∈N, *π* > 0, and as *ν* → ∞, one has

$$\begin{array}{c} \Omega_{b}\left(T\left(\upsilon \right),\upsilon ,\pi \right)\to 1*1*1=1. \end{array}$$

By (FB2), one gets T(υ)=υ. Again,

$$\begin{array}{cc} \Omega_{b}\left(\upsilon ,S\left(\upsilon \right),\pi \right) & =\Omega_{b}\left(T\left(\upsilon \right),S\left(\upsilon \right),\pi \right)\\ & \geq \Omega_{b}\left(\upsilon ,\upsilon ,\frac{\pi }{\sigma }\right)=1. \end{array}$$

Therefore, S(υ)=υ. Hence *υ* is CFP of T and S. Let *ρ* ∈ Φ ∩ *Γ* be another fixed point of T and S. Then

$$\begin{array}{c} \Omega_{b}\left(\upsilon ,\rho ,\pi \right)=\Omega_{b}\left(T\left(\upsilon \right),S\left(\rho \right),\pi \right)\geq \Omega_{b}\left(\upsilon ,\rho ,\frac{\pi }{\sigma }\right), \end{array}$$

for *σ* ∈ (0, 1) and ∀ *π* > 0. Therefore *υ* = *ρ*.

**Example 2.2**. *Suppose that* Φ = {0, 1, 2, 7} *and* $\Gamma =\{0,\frac{1}{3},\frac{1}{2},3\}$. *Define a continuous π-norm as* r*℧=min{r,℧} *and* $\Omega_{b}\left(\varphi ,\varpi ,\pi \right)={\text{exp}}^{-}\;\frac{{\left|\varphi -\varpi \right|}^{2}}{\pi }\;\forall \;\pi >0,\varphi \in \Phi$, *ϖ* ∈ *Γ*. *Then* $\left(\Phi ,\Gamma ,\Omega_{b},*\right)$ *is a complete BFBMS. Consider the mappings* T,S:Φ∪Γ→Φ∪Γ *s.t*.

$$\begin{array}{c} T\left(\varphi \right)=\{\begin{array}{cc} \frac{1}{3}, & if\;\varphi \in \{7,2\}\text{,}\\ 0, & if\;\varphi \in \{0,\frac{1}{3},\frac{1}{2},1,3\}, \end{array} \end{array}$$

*and*

$$\begin{array}{c} S\left(\varphi \right)=\{\begin{array}{cc} \frac{1}{2}, & if\;\varphi \in \{7,2\}\text{,}\\ 0, & if\;\varphi \in \{0,\frac{1}{3},\frac{1}{2},1,3\}. \end{array} \end{array}$$

*Now, suppose that* $\sigma =\frac{1}{2}$, *then* ∀ *π* > 0, *we have*

*Case 1: Let φ* ∈ {7, 2} *and ϖ* ∈ {7, 2}, *then*
  $$\begin{array}{cc} \Omega_{b}\left(T\left(\varphi \right),S\left(\varpi \right),\sigma \pi \right) & =\Omega_{b}(\frac{1}{3},\frac{1}{2},\sigma \pi )\\ & =\frac{\sigma \pi }{\sigma \pi +|\frac{1}{3}-\frac{1}{2}{|}^{2}}\\ & \geq \frac{\pi }{{\pi +|\varphi -\varpi |}^{2}}\\ & =\Omega_{b}\left(\varphi ,\varpi ,\pi \right). \end{array}$$
*Case 2: If φ* ∈ {7, 2} *and* $\varpi \in \{0,\frac{1}{3},\frac{1}{2},1,3\}$, *then*
  $$\begin{array}{cc} \Omega_{b}\left(T\left(\varphi \right),S\left(\varpi \right),\sigma \pi \right) & =\Omega_{b}(\frac{1}{3},0,\sigma \pi )\\ & =\frac{\sigma \pi }{\sigma \pi +|\frac{1}{3}{|}^{2}}\\ & \geq \frac{\pi }{{\pi +|\varphi -\varpi |}^{2}}\\ & =\Omega_{b}\left(\varphi ,\varpi ,\pi \right). \end{array}$$
*Case 3: For* $\varphi \in \{0,\frac{1}{3},\frac{1}{2},1,3\}$ *and ϖ* ∈ {7, 2}, *then*
  $$\begin{array}{cc} \Omega_{b}\left(T\left(\varphi \right),S\left(\varpi \right),\sigma \pi \right) & =\Omega_{b}\left(0,\frac{1}{2},\sigma \pi \right)\\ & =\frac{\sigma \pi }{\sigma \pi +|\frac{1}{2}{|}^{2}}\\ & \geq \frac{\pi }{{\pi +|\varphi -\varpi |}^{2}}\\ & =\Omega_{b}\left(\varphi ,\varpi ,\pi \right). \end{array}$$
*Case 4: Whenever* $\varphi \in \{0,\frac{1}{3},\frac{1}{2},1,3\}$ *and* $\varpi \in \{0,\frac{1}{3},\frac{1}{2},1,3\}$ , *then*
  $$\begin{array}{cc} \Omega_{b}\left(T\left(\varphi \right),S\left(\varpi \right),\sigma \pi \right) & =\Omega_{b}\left(0,0,\sigma \pi \right)\\ & =1\\ & \geq \frac{\pi }{{\pi +|\varphi -\varpi |}^{2}}\\ & =\Omega_{b}\left(\varphi ,\varpi ,\pi \right). \end{array}$$
  *Therefore, axiom (ii) of Theorem 2.2 also fulfills by* T *and* S. *By Theorem 2.2, we get* T *and* S *have a UCFP, i.e*., *φ* = 0.

An increasing function *Ω* : (0, 1] → (0, 1] for weak B-contraction was defined by Mihet [20], such that lim_*ν*→∞_ *Ω*^*ν*^(*σ*) = 1 and *Ω*(*σ*) ≥ *σ* ∀ *σ* ∈ (0, 1]. We are currently presenting the result as well.

**Theorem 2.3**. *Let* $\left(\Phi ,\Gamma ,\Omega_{b},℧,*\right)$ *be a BFBMS and* T,S:Φ∪Γ→Φ∪Γ *be a mappings satisfying*

*(i)* T(Φ)⊆Φ , S(Φ)⊆Φ *and* T(Γ)⊆Γ, S(Γ)⊆Γ;
*(ii) For φ* ∈ Φ, *ϖ* ∈ *Γ and* $\pi >0,\Omega_{b}\left(\varphi ,\varpi ,\pi \right)>0\Rightarrow \Omega_{b}\left(T\left(\varphi \right),S\left(\varpi \right),\pi \right)\geq \Omega \left(\Omega_{b}\left(\varphi ,\varpi ,\pi \right)\right)$.

*Then* T *and* S *have a CFP*.

*Proof*. Fix *φ*_0_ ∈ Φ and *ϖ*_0_ ∈ *Γ* and assume that $T\left(\varphi_{\nu }\right)=\varphi_{\nu +1}$, $S\left(\varphi_{\nu +1}\right)=\varphi_{\nu +2}$ and $T\left(\varpi_{\nu }\right)=\varpi_{\nu +1}$, $S\left(\varpi_{\nu +1}\right)=\varpi_{\nu +2}\;\forall \;\nu \in N\cup \left\{0\right\}$. Then ({*φ*_*ν*_}, {*ϖ*_*ν*_}) is a bisequence on BFBMS $\left(\Phi ,\Gamma ,\Omega_{b},℧,*\right)$. Now,

$$\begin{array}{c} \Omega_{b}\left(\varphi_{\nu },\varpi_{\nu },\pi \right)\geq \Omega^{\nu }\left(\Omega_{b}\left(\varphi_{0},\varpi_{0},\pi \right)\right), \end{array}$$ (9)

and

$$\begin{array}{c} \Omega_{b}\left(\varphi_{\nu +1},\varpi_{\nu },\pi \right)\geq \Omega^{\nu }\left(\Omega_{b}\left(\varphi_{1},\varpi_{0},\pi \right)\right). \end{array}$$ (10)

Letting *ν* < *κ*, for ν,κ∈N. Then,

$$\begin{array}{cc} \Omega_{b}\left(\varphi_{\nu },\varpi_{\kappa },\pi \right)\geq & \Omega_{b}\left(\varphi_{\nu },\varpi_{\nu },\frac{\pi }{3℧}\right)⋆\Omega_{b}\left(\varphi_{\nu +1},\varpi_{\nu },\frac{\pi }{3℧}\right)⋆\Omega_{b}\left(\varphi_{\nu +1},\varpi_{\kappa },\frac{\pi }{3℧}\right)\\ & \vdots \\ & \geq \Omega_{b}\left(\varphi_{\nu },\varpi_{\nu },\frac{\pi }{3℧}\right)⋆\Omega_{b}\left(\varphi_{\nu +1},\varpi_{\nu },\frac{\pi }{3℧}\right)⋆\cdots \\ & ⋆\Omega_{b}\left(\varphi_{\kappa -1},\varpi_{\kappa -1},\frac{\pi }{3^{\kappa -1}{℧}^{\kappa -1}}\right)⋆\Omega_{b}\left(\varphi_{\kappa },\varpi_{\kappa -1},\frac{\pi }{3^{\kappa -1}{℧}^{\kappa -1}}\right)\\ & ⋆\Omega_{b}\left(\varphi_{\kappa },\varpi_{\kappa },\frac{\pi }{3^{\kappa -1}{℧}^{\kappa -1}}\right). \end{array}$$

Consequently,

$$\begin{array}{cc} \Omega_{b}\left(\varphi_{\nu },\varpi_{\kappa },\pi \right) & \geq \Omega^{\nu }\left(\Omega_{b}\left(\varphi_{0},\varpi_{0},\frac{\pi }{3℧}\right)\right)⋆\Omega^{\nu }\left(\Omega_{b}\left(\varphi_{1},\varpi_{0},\frac{\pi }{3℧}\right)\right)\\ & ⋆\cdots \cdots ⋆\Omega^{\kappa }\left(\Omega_{b}\left(\varphi_{0},\varpi_{0},\frac{\pi }{3^{\kappa -1}{℧}^{\kappa -1}}\right)\right). \end{array}$$

Letting *ν*, *κ* → ∞, we have $\Omega_{b}\left(\varphi_{\nu },\varpi_{\kappa },\pi \right)\to 1\;\forall \;\pi >0$. Thus, the bisequence ({*φ*_*ν*_}, {*ϖ*_*ν*_}) is a Cauchy bisequence. Since $\left(\Phi ,\Gamma ,\Omega_{b},℧,*\right)$ is a complete, the bisequence ({*φ*_*ν*_}, {*ϖ*_*ν*_}) is a biconvergent. Therefore, the bisequence ({*φ*_*ν*_}, {*ϖ*_*ν*_}) is biconvergent then

$$\begin{array}{c} \left\{\varphi_{\nu }\right\}\to \upsilon \;\;\text{and}\;\;\left\{\varpi_{\nu }\right\}\to \upsilon \;\forall \;\upsilon \in \Phi \cap \Gamma . \end{array}$$

From (FB4),

$$\begin{array}{cc} \Omega_{b}\left(T\left(\upsilon \right),\upsilon ,\pi \right) & \geq \Omega_{b}\left(T\left(\upsilon \right),\varpi_{\nu +1},\frac{\pi }{3℧}\right)⋆\Omega_{b}\left(\varphi_{\nu +1},\varpi_{\nu +1},\frac{\pi }{3℧}\right)*\Omega_{b}\left(\varphi_{\nu +1},\upsilon ,\frac{\pi }{3℧}\right)\\ & =\Omega_{b}\left(T\left(\upsilon \right),S\left(\varpi_{\nu }\right),\frac{\pi }{3℧}\right)⋆\Omega_{b}\left(\varphi_{\nu +1},\varpi_{\nu +1},\frac{\pi }{3℧}\right)*\Omega_{b}\left(\varphi_{\nu +1},\upsilon ,\frac{\pi }{3℧}\right)\\ & \geq \Omega \left(\Omega_{b}\left(\upsilon ,\varpi_{\nu },\frac{\pi }{3℧}\right)\right)⋆\Omega_{b}\left(\varphi_{\nu +1},\varpi_{\nu +1},\frac{\pi }{3℧}\right)*\Omega_{b}\left(\varphi_{\nu +1},\upsilon ,\frac{\pi }{3℧}\right), \end{array}$$

for all ν∈N and *π* > 0. Letting *ν* → ∞, we have

$$\begin{array}{c} \Omega_{b}\left(T\left(\upsilon \right),\upsilon ,\pi \right)\to 1*1*1=1. \end{array}$$

From (FB2), we get T(υ)=υ. Again,

$$\begin{array}{cc} \Omega_{b}\left(\upsilon ,S\left(\upsilon \right),\pi \right) & =\Omega_{b}\left(T\left(\upsilon \right),S\left(\upsilon \right),\pi \right)\\ & \geq \Omega (\Omega_{b}\left(\upsilon ,\upsilon ,\pi \right)). \end{array}$$

Therefore, S(υ)=υ. Hence *υ* is a CFP of T and S.

**Example 2.3**. *Let* Φ={2,4,5,6},Γ={1,2},i*b=ib∀i,b∈[0,1] *and*

$$\begin{array}{c} \Omega_{b}\left(\varphi ,\varpi ,\pi \right)=\frac{{\left(\text{min}\;\left\{\varphi ,\varpi \right\}\right)}^{2}+\pi }{{\left(\text{max}\;\left\{\varphi ,\varpi \right\}\right)}^{2}+\pi }\;\forall \;\varphi \in \Phi ,\varpi \in \Gamma \;and\;\forall \;\pi >0. \end{array}$$

*Then*, $\left(\Phi ,\Gamma ,\Omega_{b},℧,*\right)$ *is a complete BFBMS. Now, define Ω* : (0, 1] → (0, 1] *s.t*. $\Omega \left(\sigma \right)=\sqrt{\sigma }$. *Clearly*, $\Omega \left(\sigma \right)=\sqrt{\sigma }$ *satisfies conditions of Ω function. Consider the mappings* T,S:Φ∪Γ→Φ∪Γ *given by* T(2)=T(4)=T(1)=2,T(5)=T(6)=4 *and* S(2)=S(5)=S(6)=2,S(4)=S(1)=4. *Then, every need stated in Theorem 2.3 is met. The CFP of* T *and* S *is φ* = 2.

In the end, we investigate CFP on BFBMS with weak B-contraction and contravariant mapping.

**Theorem 2.4**. *Consider* $\left(\Phi ,\Gamma ,\Omega_{b},℧,*\right)$ *to be a BFBMS, and* T,S:Φ∪Γ→Φ∪Γ *be a mappings satisfying*

*(i)* T(Φ)⊆Γ , T(Γ)⊆Φ *and* S(Φ)⊆Γ, S(Γ)⊆Φ;
*(ii)* *For φ* ∈ Φ, *ϖ* ∈ *Γ and* $\pi >0,\Omega_{b}\left(\varphi ,\varpi ,\pi \right)>0\Rightarrow \Omega_{b}\left(T\left(\varpi \right),S\left(\varphi \right),\pi \right)\geq \Omega \left(\Omega_{b}\left(\varphi ,\varpi ,\pi \right)\right)$.

*Then*, T *and* S *have a CFP*.

*Proof*. Fix *φ*_0_ ∈ Φ and *ϖ*_0_ ∈ *Γ* and assume that $T\left(\varphi_{2\nu }\right)=\varpi_{2\nu }$, $S\left(\varphi_{2\nu +1}\right)=\varpi_{2\nu +1}$ and $T\left(\varpi_{2\nu }\right)=x{‘}_{2\nu +1}$, $S\left(\varpi_{2\nu +1}\right)=\varphi_{2\nu +2}\;\forall \;\nu \in N\cup \left\{0\right\}$. Then ({*φ*_*ν*_}, {*ϖ*_*ν*_}) is a bisequence on BFBMS $\left(\Phi ,\Gamma ,\Omega_{b},*\right)$. Now,

$$\begin{array}{c} \Omega_{b}\left(\varphi_{2\nu +1},\varpi_{2\nu +1},\pi \right)\geq \Omega^{4\nu +2}\left(\Omega_{b}\left(\varphi_{0},\varpi_{0},\pi \right)\right), \end{array}$$ (11)

and

$$\begin{array}{c} \Omega_{b}\left(\varphi_{2\nu +1},\varpi_{2\nu },\pi \right)\geq \Omega^{4\nu +1}\left(\Omega_{b}\left(\varphi_{1},\varpi_{0},\pi \right)\right). \end{array}$$ (12)

Letting *ν* < *κ*, for ν,κ∈N. Then,

$$\begin{array}{cc} \Omega_{b}\left(\varphi_{\nu },\varpi_{\kappa },\pi \right)\geq & \Omega_{b}\left(\varphi_{\nu },\varpi_{\nu },\frac{\pi }{3℧}\right)⋆\Omega_{b}\left(\varphi_{\nu +1},\varpi_{\nu },\frac{\pi }{3℧}\right)⋆\Omega_{b}\left(\varphi_{\nu +1},\varpi_{\kappa },\frac{\pi }{3℧}\right)\\ & \vdots \\ & \geq \Omega_{b}\left(\varphi_{\nu },\varpi_{\nu },\frac{\pi }{3℧}\right)⋆\Omega_{b}\left(\varphi_{\nu +1},\varpi_{\nu },\frac{\pi }{3℧}\right)⋆\cdots \\ & ⋆\Omega_{b}\left(\varphi_{\kappa -1},\varpi_{\kappa -1},\frac{\pi }{3^{\kappa -1}{℧}^{\kappa -1}}\right)⋆\Omega_{b}\left(\varphi_{\kappa },\varpi_{\kappa -1},\frac{\pi }{3^{\kappa -1}{℧}^{\kappa -1}}\right)\\ & ⋆\Omega_{b}\left(\varphi_{\kappa },\varpi_{\kappa },\frac{\pi }{3^{\kappa -1}{℧}^{\kappa -1}}\right). \end{array}$$

Consequently,

$$\begin{array}{cc} \Omega_{b}\left(\varphi_{\nu },\varpi_{\kappa },\pi \right) & \geq \Omega^{2\nu }\left(\Omega_{b}\left(\varphi_{0},\varpi_{0},\frac{\pi }{3℧}\right)\right)⋆\Omega^{2\nu +1}\left(\Omega_{b}\left(\varphi_{1},\varpi_{0},\frac{\pi }{3℧}\right)\right)\\ & ⋆\cdots \cdots ⋆\Omega^{2\kappa }\left(\Omega_{b}\left(\varphi_{0},\varpi_{0},\frac{\pi }{3^{\kappa -1}{℧}^{\kappa -1}}\right)\right). \end{array}$$

Letting *ν*, *κ* → ∞, we have $\Omega_{b}\left(\varphi_{\nu },\varpi_{\kappa },\pi \right)\to 1\;\forall \;\pi >0$. Thus, the bisequence ({*φ*_*ν*_}, {*ϖ*_*ν*_}) is a Cauchy bisequence. Since $\left(\Phi ,\Gamma ,\Omega_{b},℧,*\right)$ is a complete, the bisequence ({*φ*_*ν*_}, {*ϖ*_*ν*_}) is a biconvergent. Therefore, the bisequence ({*φ*_*ν*_}, {*ϖ*_*ν*_}) is biconvergent then

$$\begin{array}{c} \left\{\varphi_{\nu }\right\}\to \upsilon \;\;\text{and}\;\;\left\{\varpi_{\nu }\right\}\to \upsilon \;\forall \;\upsilon \in \Phi \cap \Gamma . \end{array}$$

From (FB4),

$$\begin{array}{cc} \Omega_{b}\left(T\left(\upsilon \right),\upsilon ,\pi \right) & \geq \Omega_{b}\left(T\left(\upsilon \right),\varpi_{\nu +1},\frac{\pi }{3℧}\right)⋆\Omega_{b}\left(\varphi_{\nu +1},\varpi_{\nu +1},\frac{\pi }{3℧}\right)*\Omega_{b}\left(\varphi_{\nu +1},\upsilon ,\frac{\pi }{3℧}\right)\\ & =\Omega_{b}\left(T\left(\upsilon \right),S\left(\varphi_{\nu +1}\right),\frac{\pi }{3℧}\right)⋆\Omega_{b}\left(T\varpi_{\nu },S\varphi_{\nu +1},\frac{\pi }{3℧}\right)⋆\Omega_{b}\left(\varphi_{\nu +1},\upsilon ,\frac{\pi }{3℧}\right)\\ & \geq \Omega \left(\Omega_{b}\left(\upsilon ,\varphi_{\nu +1},\frac{\pi }{3℧}\right)\right)⋆\Omega \left(\Omega_{b}\left(\varpi_{\nu },\varphi_{\nu +1},\frac{\pi }{3℧}\right)\right)*\Omega_{b}\left(\varphi_{\nu +1},\upsilon ,\frac{\pi }{3℧}\right), \end{array}$$

∀ν∈N, *π* > 0, and as *ν* → ∞, one has

$$\begin{array}{c} \Omega_{b}\left(T\left(\upsilon \right),\upsilon ,\pi \right)\to 1*1*1=1. \end{array}$$

By (FB2), one gets T(υ)=υ. Again,

$$\begin{array}{cc} \Omega_{b}\left(\upsilon ,S\left(\upsilon \right),\pi \right) & =\Omega_{b}\left(T\left(\upsilon \right),S\left(\upsilon \right),\pi \right)\\ & \geq \Omega (\Omega_{b}\left(\upsilon ,\upsilon ,\pi \right))=1. \end{array}$$

Therefore, S(υ)=υ. Hence *υ* is a CFP of T and S.

## 3 Application to integral equations

In this section, we use the terms and conditions of the Theorem 2.1 by studying a solution of integral equations.

**Theorem 3.1**. *Consider the following coupled integral equations*

$$\begin{array}{c} \left\{ \begin{array}{c} \varphi \left(\omega \right)=b\left(\omega \right)+\int_{\Lambda_{1}\cup \Lambda_{2}}G_{1}\left(\omega ,℧,\varphi \left(℧\right)\right)d℧,\;\;\omega \in \Lambda_{1}\cup \Lambda_{2},\\ \varphi \left(\omega \right)=b\left(\omega \right)+\int_{\Lambda_{1}\cup \Lambda_{2}}G_{2}\left(\omega ,℧,\varphi \left(℧\right)\right)d℧,\;\;\omega \in \Lambda_{1}\cup \Lambda_{2}, \end{array}\right. \end{array}$$ (13)

*where Λ*_1_ ∪ *Λ*_2_ *is a Lebesgue measurable set. Consider*

*(T1)* $G_{1},G_{2}:\left(\Lambda_{1}^{2}\cup \Lambda_{2}^{2}\right)\times \left[0,\infty \right)\to \left[0,\infty \right)$ *and b* ∈ *L*^∞^(*Λ*_1_) ∪ *L*^∞^(*Λ*_2_),
*(T2)* *There exists a continuous function* $\theta :\Lambda_{1}^{2}\cup \Lambda_{2}^{2}\to \left[0,\infty \right)$ , *and σ* ∈ (0, 1) *s.t*.
  $$\begin{array}{c} |G_{1}\left(\omega ,℧,\varphi \left(℧\right)\right)-G_{2}\left(\omega ,℧,\varpi \left(℧\right)\right)|\leq \sqrt{\sigma \theta (\omega ,℧)}(|\varphi \left(\omega \right)-\varpi \left(\omega \right)|), \end{array}$$
  *for* $\omega ,℧\in \Lambda_{1}^{2}\cup \Lambda_{2}^{2}$,
*(T3)* ${\text{sup}}_{\omega \in \Lambda_{1}\cup \Lambda_{2}}\int_{\Lambda_{1}\cup \Lambda_{2}}\theta \left(\omega ,℧\right)\;d℧\leq 1$ .

*Then, the coupled integral equations* (13) *concede a common solution in L*^∞^(*Λ*_1_) ∪ *L*^∞^(*Λ*_2_).

*Proof*. Let Φ = *L*^∞^(*Λ*_1_), and *Γ* = *L*^∞^(*Λ*_2_) be two normed linear spaces, where *Λ*_1_, *Λ*_2_ are Lebesgue measurable sets and *m*(*Λ*_1_ ∪ *Λ*_2_) < ∞.

Define $\Omega_{b}:\Phi \times \Gamma \times \left(0,\infty \right)\to \left[0,1\right]$ by

$$\begin{array}{c} \Omega_{b}\left(\varphi ,\varpi ,\pi \right)=e^{-\frac{{\text{sup}}_{\omega \in \Lambda_{1}\cup \Lambda_{2}}{\left|\varphi \left(\omega \right)-\varpi \left(\omega \right)\right|}^{2}}{\pi }}. \end{array}$$

for all *φ* ∈ Φ, *ϖ* ∈ *Γ*. Then, $\left(\Phi ,\Gamma ,\Omega_{b},⋆\right)$ is a complete BFBMS.

Define $T,S:L^{\infty }\left(\Lambda_{1}\right)\cup L^{\infty }\left(\Lambda_{2}\right)\to L^{\infty }\left(\Lambda_{1}\right)\cup L^{\infty }\left(\Lambda_{2}\right)$ by

$$\begin{array}{c} T\left(\varphi \left(\omega \right)\right)=b\left(\omega \right)+\int_{\Lambda_{1}\cup \Lambda_{2}}G_{1}\left(\omega ,℧,\varphi \left(℧\right)\right)d℧,\;\;\omega \in \Lambda_{1}\cup \Lambda_{2}, \end{array}$$

$$\begin{array}{c} S\left(\varphi \left(\omega \right)\right)=b\left(\omega \right)+\int_{\Lambda_{1}\cup \Lambda_{2}}G_{2}\left(\omega ,℧,\varphi \left(℧\right)\right)d℧,\;\;\omega \in \Lambda_{1}\cup \Lambda_{2}. \end{array}$$

Now,

$$\begin{array}{cc} \Omega_{b}\left(T\varphi \left(\omega \right),S\varpi \left(\omega \right),\sigma \pi \right) & =e^{-{\text{sup}}_{\omega \in \Lambda_{1}\cup \Lambda_{2}}\frac{{\left|T\varphi \left(\omega \right)-S\varpi \left(\omega \right)\right|}^{2}}{\sigma \pi }}\\ & =e^{-{\text{sup}}_{\omega \in \Lambda_{1}\cup \Lambda_{2}}\frac{|b\left(\omega \right)+\int_{\Lambda_{1}\cup \Lambda_{2}}G_{1}\left(\omega ,℧,\varphi \left(℧\right)\right)d℧-b\left(\omega \right)-\int_{\Lambda_{1}\cup \Lambda_{2}}G_{2}\left(\omega ,℧,\varphi \left(℧\right)\right)d℧{)|}^{2}}{\sigma \pi }}\\ & \geq e^{-{\text{sup}}_{\omega \in \Lambda_{1}\cup \Lambda_{2}}\frac{\int_{\Lambda_{1}\cup \Lambda_{2}}{\left|G_{1}\left(\omega ,℧,\varphi \left(℧\right)\right)-G_{2}\left(\omega ,℧,\varpi \left(℧\right)\right)\right|}^{2}d℧}{\sigma \pi }}\\ & \geq e^{-{\text{sup}}_{\omega \in \Lambda_{1}\cup \Lambda_{2}}\frac{\int_{\Lambda_{1}\cup \Lambda_{2}}{\sigma \theta \left(\omega ,℧\right)(|\varphi \left(\omega \right)-\varpi \left(\omega \right)|}^{2})d℧}{\sigma \pi }}\\ & \geq e^{-{\text{sup}}_{\omega \in \Lambda_{1}\cup \Lambda_{2}}\frac{\int_{\Lambda_{1}\cup \Lambda_{2}}{\sigma \theta \left(\omega ,℧\right)(|\varphi \left(\omega \right)-\varpi \left(\omega \right)|}^{2})d℧}{\sigma \pi }}\\ & \geq e^{-{\text{sup}}_{\omega \in \Lambda_{1}\cup \Lambda_{2}}\frac{{\left|\varphi \left(\omega \right)-\varpi \left(\omega \right)\right|}^{2}}{\pi }}\\ & =\Omega_{b}\left(\varphi ,\varpi ,\pi \right). \end{array}$$

Thus, all the hypothesis of the Theorem 2.1 are satisfied. Therefore, the coupled integral equations (13) possesses one common solution.

## 4 Application to fractional differential equations

Physical systems with continuous distributions or interactions can be modeled and investigated with the help of fractional differential equations (FDEs). They are often used in engineering research to extract relationships between numbers or to provide a more detailed description of phenomena than differential equations alone can. They provide a framework to grasp various engineering systems’ intricate interactions and behaviors. Implicit fractional differential equations (IFDEs) have various potential uses in engineering research. We demonstrate that there is a single solution to the IFDE in this section. In engineering, differential equations of this type are commonly used. They are necessary for material science research, heat exchange, field magnetic assessment for radars, structural evaluation, mechanisms for control, digital circuits assessment, mechanical design fatigue and circulation of fluids simulation, and data processing operations. They are also useful in geophysics, non-destructive testing, medical imaging, and inverse issues related to ophthalmology and acoustically for propagation of waves and diffraction studies. These equations provide a flexible framework for understanding and assessing continuous interactions and distributions in various engineering fields [21–24]. Younis and Abdou [25] innovative method by combining concepts from graph mappings, Kannan mappings, and fuzzy contractions to produce a completely new idea known as Kannan-graph-fuzzy contraction and applications to engineering science. For more details, we refer readers to these works [26, 27]. In what follows, we prove the uniqueness of solution for the following fractional differential equations in the sense of Caputo derivative. For more details see this work [28].

$$\begin{array}{c} D_{0+}^{\delta }\varphi \left(\omega \right)+g_{1}\left(\omega ,\varphi \left(\omega \right)\right)=0,\;0<\omega <1,\\ D_{0+}^{\delta }\varphi \left(\omega \right)+g_{2}\left(\omega ,\varphi \left(\omega \right)\right)=0,\;0<\omega <1, \end{array}$$ (14)

under the boundary value conditions

$$\varphi \left(0\right)+\varphi^{\prime }\left(0\right)=0,\;\varphi \left(1\right)+\varphi^{\prime }\left(1\right)=0,$$

where, 1 < *δ* ≤ 2, $g_{1},g_{2}:\left[0,1\right]\times \left[0,\infty \right)\to \left[0,\infty \right)$ are continuous functions. Let Φ=(C[0,1],[0,∞))={z:[0,1]→[0,∞):zisacontinuousfunction}, and Γ=(C[0,1],(-∞,0])={z:[0,1]→(-∞,0]:zisacontinuousfunction}. Define $\Omega_{b}:\Phi \times \Gamma \times \left(0,\infty \right)\to \left[0,1\right]$ by

$$\begin{array}{c} \Omega_{b}\left(\varphi ,\varpi ,\pi \right)=e^{-\frac{{\text{sup}}_{\omega \in \Lambda_{1}\cup \Lambda_{2}}{\left|\varphi \left(\omega \right)-\varpi \left(\omega \right)\right|}^{2}}{\pi }}, \end{array}$$

where i*b=ib. Note that *φ* ∈ Φ ∪ *Γ* solves (14) and whenever *φ* ∈ Φ ∪ *Γ* is the solution of

$$\begin{array}{cc} \varphi (\omega )= & \frac{1}{\Gamma (\delta )}\int_{0}^{1}{\left(1-\lambda \right)}^{\delta -1}\left(1-\omega \right)g_{1}\left(\lambda ,\varphi \left(\lambda \right)\right)d\lambda \\ & +\frac{1}{\Gamma (\delta -1)}\int_{0}^{1}{\left(1-\lambda \right)}^{\delta -2}\left(1-\omega \right)g_{1}\left(\lambda ,\varphi \left(\lambda \right)\right)d\lambda \\ & +\frac{1}{\Gamma (\delta )}\int_{0}^{\omega }{\left(\omega -\lambda \right)}^{\delta -1}g_{2}\left(\lambda ,\varphi \left(\lambda \right)\right)d\lambda ,\\ \varphi (\omega )= & \frac{1}{\Gamma (\delta )}\int_{0}^{1}{\left(1-\lambda \right)}^{\delta -1}\left(1-\omega \right)g_{2}\left(\lambda ,\varphi \left(\lambda \right)\right)d\lambda \\ & +\frac{1}{\Gamma (\delta -1)}\int_{0}^{1}{\left(1-\lambda \right)}^{\delta -2}\left(1-\omega \right)g_{2}\left(\lambda ,\varphi \left(\lambda \right)\right)d\lambda \\ & +\frac{1}{\Gamma (\delta )}\int_{0}^{\omega }{\left(\omega -\lambda \right)}^{\delta -1}g_{2}\left(\lambda ,\varphi \left(\lambda \right)\right)d\lambda . \end{array}$$

**Theorem 4.1**. *Consider the operators* T,S:Φ∪Γ→Φ∪Γ *are given by*:

$$\begin{array}{cc} T\varphi (\omega )= & \frac{1}{\Gamma (\delta )}\int_{0}^{1}{\left(1-\lambda \right)}^{\delta -1}\left(1-\omega \right)g_{1}\left(\lambda ,\varphi \left(\lambda \right)\right)d\lambda \\ & +\frac{1}{\Gamma (\delta -1)}\int_{0}^{1}{\left(1-\lambda \right)}^{\delta -2}\left(1-\omega \right)g_{1}\left(\lambda ,\varphi \left(\lambda \right)\right)d\lambda \\ & +\frac{1}{\Gamma (\delta )}\int_{0}^{\omega }{\left(\omega -\lambda \right)}^{\delta -1}g_{1}\left(\lambda ,\varphi \left(\lambda \right)\right)d\lambda ,\\ S\varphi (\omega )= & \frac{1}{\Gamma (\delta )}\int_{0}^{1}{\left(1-\lambda \right)}^{\delta -1}\left(1-\omega \right)g_{2}\left(\lambda ,\varphi \left(\lambda \right)\right)d\lambda \\ & +\frac{1}{\Gamma (\delta -1)}\int_{0}^{1}{\left(1-\lambda \right)}^{\delta -2}\left(1-\omega \right)g_{2}\left(\lambda ,\varphi \left(\lambda \right)\right)d\lambda \\ & +\frac{1}{\Gamma (\delta )}\int_{0}^{\omega }{\left(\omega -\lambda \right)}^{\delta -1}g_{2}\left(\lambda ,\varphi \left(\lambda \right)\right)d\lambda . \end{array}$$

*Suppose the following conditions hold*:

*(i) for all φ* ∈ Φ, *ϖ* ∈ *Γ and* $g_{1},g_{2}:\left[0,1\right]\times \left[0,\infty \right)\to \left[0,\infty \right)$, *satisfies*
  $$\begin{array}{c} |g_{1}\left(\lambda ,\varphi \left(\lambda \right)\right)-g_{2}\left(\lambda ,\varpi \left(\lambda \right)\right)|\leq \sigma^{\frac{1}{2}}\left|\varphi \left(\lambda \right)-\varpi \left(\lambda \right)\right|, \end{array}$$
*(ii)*
$$\begin{array}{c} \underset{\omega \in (0,1)}{\text{sup}}\;|\frac{1-\omega }{\Gamma (\delta +1)}+\frac{1-\omega }{\Gamma (\delta )}+\frac{\omega^{\delta }}{\Gamma (\delta +1)}{|}^{2}=\eta <1. \end{array}$$

*Then, the system* (14) *have a unique common solution*.

*Proof*. Assume that *φ* ∈ Φ, *ϖ* ∈ *Γ*, and consider that

$$\begin{array}{cc} {\left|T\varphi \left(\omega \right)-S\varpi \left(\omega \right)\right|}^{2}= & |\frac{1}{\Gamma (\delta )}\int_{0}^{1}{\left(1-\lambda \right)}^{\delta -1}\left(1-\omega \right)\left(g_{1}\left(\lambda ,\varphi \left(\lambda \right)-g_{2}\left(\lambda ,\varpi \left(\lambda \right)\right)\right)\right)d\lambda \\ & +\frac{1}{\Gamma (\delta -1)}\int_{0}^{1}{\left(1-\lambda \right)}^{\delta -2}\left(1-\omega \right)\left(g_{1}\left(\lambda ,\varphi \left(\lambda \right)-g_{2}\left(\lambda ,\varpi \left(\lambda \right)\right)\right)\right)d\lambda \\ & +\frac{1}{\Gamma (\delta )}\int_{0}^{\omega }{\left(\omega -\lambda \right)}^{\delta -1}\left(g_{1}\left(\lambda ,\varphi \left(\lambda \right)-g_{2}\left(\lambda ,\varpi \left(\lambda \right)\right)\right)\right)d\lambda {|}^{2}\\ & \leq (\frac{1}{\Gamma (\delta )}\int_{0}^{1}{\left(1-\lambda \right)}^{\delta -1}\left(1-\omega \right)|\left(g_{1}\left(\lambda ,\varphi \left(\lambda \right)-g_{2}\left(\lambda ,\varpi \left(\lambda \right)\right)\right)\right)|d\lambda \\ & +\frac{1}{\Gamma (\delta -1)}\int_{0}^{1}{\left(1-\lambda \right)}^{\delta -2}\left(1-\omega \right)|\left(g_{1}\left(\lambda ,\varphi \left(\lambda \right)-g_{2}\left(\lambda ,\varpi \left(\lambda \right)\right)\right)\right)|d\lambda \\ & +\frac{1}{\Gamma (\delta )}\int_{0}^{\omega }{\left(\omega -\lambda \right)}^{\delta -1}|\left(g_{1}\left(\lambda ,\varphi \left(\lambda \right)-g_{2}\left(\lambda ,\varpi \left(\lambda \right)\right)\right)\right)|d\lambda {)}^{2}\\ & \leq (\frac{1}{\Gamma (\delta )}\int_{0}^{1}{\left(1-\lambda \right)}^{\delta -1}\left(1-\omega \right)\sigma^{\frac{1}{2}}\left|\varphi \left(\lambda \right)-\varpi \left(\lambda \right)\right|d\lambda \\ & +\frac{1}{\Gamma (\delta -1)}\int_{0}^{1}{\left(1-\lambda \right)}^{\delta -2}\left(1-\omega \right)\sigma^{\frac{1}{2}}\left|\varphi \left(\lambda \right)-\varpi \left(\lambda \right)\right|d\lambda \\ & +\frac{1}{\Gamma (\delta )}\int_{0}^{\omega }{\left(\omega -\lambda \right)}^{\delta -1}\sigma^{\frac{1}{2}}\left|\varphi \left(\lambda \right)-\varpi \left(\lambda \right)\right|d\lambda {)}^{2}\\ & ={\sigma |\varphi \left(\omega \right)-\varpi \left(\omega \right)|}^{2}(\frac{1}{\Gamma (\delta )}\int_{0}^{1}{\left(1-\lambda \right)}^{\delta -1}\left(1-\omega \right)d\lambda \\ & +\frac{1}{\Gamma (\delta -1)}\int_{0}^{1}{\left(1-\lambda \right)}^{\delta -2}\left(1-\omega \right)d\lambda +\frac{1}{\Gamma (\delta )}\int_{0}^{\omega }{\left(\omega -\lambda \right)}^{\delta -1}d\lambda {)}^{2}\\ & ={\sigma |\varphi \left(\omega \right)-\varpi \left(\omega \right)|}^{2}{\left(\frac{1-\omega }{\Gamma (\delta +1)}+\frac{1-\omega }{\Gamma (\delta )}+\frac{\omega^{\delta }}{\Gamma (\delta +1)}\right)}^{2}\\ & \leq {\sigma |\varphi \left(\omega \right)-\varpi \left(\omega \right)|}^{2}\underset{\omega \in (0,1)}{\text{sup}}\;{\left(\frac{1-\omega }{\Gamma (\delta +1)}+\frac{1-\omega }{\Gamma (\delta )}+\frac{\omega^{\delta }}{\Gamma (\delta +1)}\right)}^{2}\\ & ={\eta \sigma |\varphi \left(\omega \right)-\varpi \left(\omega \right)|}^{2}\\ & \leq {\sigma |\varphi \left(\omega \right)-\varpi \left(\omega \right)|}^{2}. \end{array}$$

So, we have

$$\begin{array}{c} |T\varphi \left(\omega \right)-S\varpi \left(\omega \right){|}^{2}\leq \sigma {\left|\varphi \left(\omega \right)-\varpi \left(\omega \right)\right|}^{2}, \end{array}$$

i.e.,

$$\begin{array}{cc} -\frac{{\text{sup}}_{\omega \in [0,1]}|T\varphi \left(\omega \right)-S\varpi \left(\omega \right){|}^{2}}{\sigma \pi } & \geq -\frac{{\text{sup}}_{\omega \in [0,1]}{\left|\varphi \left(\omega \right)-\varpi \left(\omega \right)\right|}^{2}}{\pi }\\ \text{exp}\;(-\frac{{\text{sup}}_{\omega \in [0,1]}|T\varphi \left(\omega \right)-S\varpi \left(\omega \right){|}^{2}}{\sigma \pi }) & \geq \;\text{exp}\;(-\frac{{\text{sup}}_{\omega \in [0,1]}{\left|\varphi \left(\omega \right)-\varpi \left(\omega \right)\right|}^{2}}{\pi }), \end{array}$$

thus, we have

$$\begin{array}{c} \Omega_{b}\left(T\varphi \left(\omega \right),S\varpi \left(\omega \right),\sigma \pi \right)\geq \Omega_{b}\left(\varphi \left(\omega \right),\varpi \left(\omega \right),\pi \right). \end{array}$$

Therefore, all the terms and conditions of the Theorem 2.1 has been verified and as a result, there exists a common solution for the Caputo fractional system (14).

## 5 Conclusion

In the present paper, we study novel CFP theorems on BFBMS under both covariant and contravariant mappings, and we also provide illustrative examples. Additionally, CFP was studied under weak B-contraction mapping, and a helpful example is provided. Lastly, we examined the existence and uniqueness of the solution for fractional differential and integral equations in order to assess the efficacy of our findings.

For the future, authors of the work [29], proved a common coupled fixed point theorem on bipolar fuzzy metric space. It is an open problem to prove the common coupled fixed point theorems on BFBMS.
